# Depression and social frailty in elderly patients receiving maintenance hemodialysis: multiple mediating roles of bidirectional social support and personal mastery

**DOI:** 10.3389/fmed.2025.1647396

**Published:** 2025-10-22

**Authors:** Neng Wang, Guoqing Wang, Xuefen Wang, Dejiao He

**Affiliations:** ^1^Nephrology Department, Renmin Hospital of Wuhan University, Wuhan, Hubei, China; ^2^Hemodialysis Center, Renmin Hospital of Wuhan University East, Wuhan, Hubei, China; ^3^Nephrology II Department, Renmin Hospital of Wuhan University, Wuhan, Hubei, China

**Keywords:** maintenance hemodialysis, depression, social frailty, social support, sense of personal mastery, mediation effect

## Abstract

**Objective:**

This study aimed to investigate the multiple mediating roles of bidirectional social support and sense of personal mastery in the relationship between depression and social frailty among elderly patients undergoing maintenance hemodialysis (MHD).

**Methods:**

A cross-sectional survey was conducted among 248 elderly MHD patients from two tertiary hospitals in Wuhan, China. Data were collected using a general information questionnaire, the Social Vulnerability Index, the Geriatric Depression Scale-5 (GDS-5), the Brief Two-Way Social Support Scale, and the Personal Mastery Scale. Structural Equation Modeling (SEM) was performed using AMOS 28.0.

**Results:**

The mean scores were as follows: depression (1.91 ± 1.67), bidirectional social support (38.49 ± 8.43), personal mastery(21.51 ± 4.95), and SVI (0.49 ± 0.19). Depression was positively correlated with social frailty (*r* = 0.716, *p* < 0.01), whereas both personal mastery and bidirectional social support were negatively correlated with social frailty (*r* = −0.721, −0.760, both *p* < 0.01). Mediation analysis indicated that bidirectional social support and sense of personal mastery partially mediated the relationship between depression and social frailty, with a total indirect effect size of 0.524, accounting for 67.96% of the total effect.

**Conclusion:**

Social frailty among elderly MHD patients is at a moderate level, with 27.02% experiencing moderate to severe social frailty. The prevalence of depressive symptoms was 53.6%. Depression significantly predicts social frailty, while bidirectional social support and personal mastery serve as partial mediators. Enhancing these psychosocial factors may help reduce the risk or severity of social frailty in this population.

## Introduction

With the global population aging and the increasing prevalence of renal diseases, Chronic Kidney Disease (CKD) has become a significant public health concern. According to statistics, about 2% of CKD patients in China enter end-stage renal disease (ESRD) every year (1). ESRD is defined as the terminal stage of various forms of CKD, characterized by irreversible kidney failure. According to the International Society of Nephrology (ISN), the number of individuals suffering from ESRD is projected to reach 14.5 million globally by 2030 (2). Maintenance hemodialysis (MHD) remains the predominant renal replacement therapy for ESRD (3), functioning to eliminate metabolic waste and prolong patient survival. In China, individuals aged 60 years and older are legally classified as elderly (4), and they comprise nearly 70% of the MHD population (5). Despite the life-prolonging benefits of dialysis, elderly MHD patients commonly experience psychological and social challenges due to comorbidities and poor treatment adherence, contributing to a five-year survival rate of less than 40% (6). Previous studies have demonstrated a close association between mental health status and patient survival rates (7). Furthermore, factors such as cognitive impairment, chronic pain, alcohol or other substance dependence, along with an elevated suicide risk, must be considered in research on this population (8–10). Consequently, promoting healthy aging and enhancing psychosocial adaptation among this population have become a healthcare priority in China (11).

Depression is a common psychiatric disorder with a notably high prevalence among patients undergoing maintenance hemodialysis. A study conducted in northern China reported that approximately 55.1% of MHD patients exhibited depressive symptoms (12). Although mental disorders severely impair survival in this population, they are frequently overlooked as risk factors (13). The underlying mechanisms include inflammation associated with uremic toxins, reduced treatment adherence, and uncertainty regarding prognosis. Furthermore, studies have shown that male gender, hypoalbuminemia, malnutrition, poor quality of life, and psychological resilience are significant predictors of mental disorders in patients with MHD (14). Social frailty refers to a condition in which individuals are at heightened risk due to persistent unmet social needs, characterized by progressive impairments in accessing social resources, maintaining social roles, engaging in social activities, and exercising self-management (15). According to the Diagnostic and Statistical Manual of Mental Disorders (DSM-5), a lack of interest or pleasure in social interactions is a core diagnostic feature of depressive disorders. Depression not only contributes to the development of social frailty (16), but also directly reduces social motivation and participation. Alleviating depressive symptoms may help patients re-engage in social life, preserve their social networks, and restore functional capacities. Nevertheless, the specific mechanisms by which depression influences social frailty remain unclear. Elucidating these pathways may assist healthcare providers in developing interventions to mitigate social frailty among elderly MHD patients.

Bidirectional social support refers to a reciprocal network in which individuals both provide and receive emotional or instrumental assistance from others (17). Social support is inversely associated with depression and may help alleviate depressive symptoms by regulating physiological stress responses, such as cortisol secretion (18). Robust social support enhances psychological wellbeing, fulfills basic social needs, improves life satisfaction, and facilitates recovery from illness (19). Personal mastery is a multidimensional psychological construct that reflects an individual’s perceived control over life circumstances and their ability to cope with and regulate life events (20). Empirical evidence suggests a negative association between depression and personal mastery (21). As a fundamental component of psychological resilience (22), personal mastery plays a critical role in enhancing cognitive flexibility, facilitating emotional recovery, and reducing the impact of stressors. Individuals with a high level of personal mastery are more likely to adopt proactive and problem-focused coping strategies, which can reshape their social perceptions, strengthen self-efficacy, and ultimately mitigate tendencies toward social withdrawal.

Structural Equation Modeling (SEM) is a multivariate statistical technique that integrates confirmatory factor analysis and path analysis (23). It enables the simultaneous examination of complex causal relationships among multiple independent and dependent variables, allowing for the estimation of both direct and indirect effects and the assessment of their relative magnitudes. Although previous research has demonstrated correlations among depression, bidirectional social support, personal mastery, and social frailty in individuals with chronic illnesses; however, the combined effects of these variables on social frailty in elderly MHD patients remain unverified. To address this gap, the present study is the first to employ SEM to investigate the multiple mediating roles of bidirectional social support and personal mastery in the relationship between depression and social frailty in elderly MHD patients. By analyzing these associations, the study aims to provide a theoretical foundation for interventions targeting depression and social frailty in this population.

**Figure 1:**
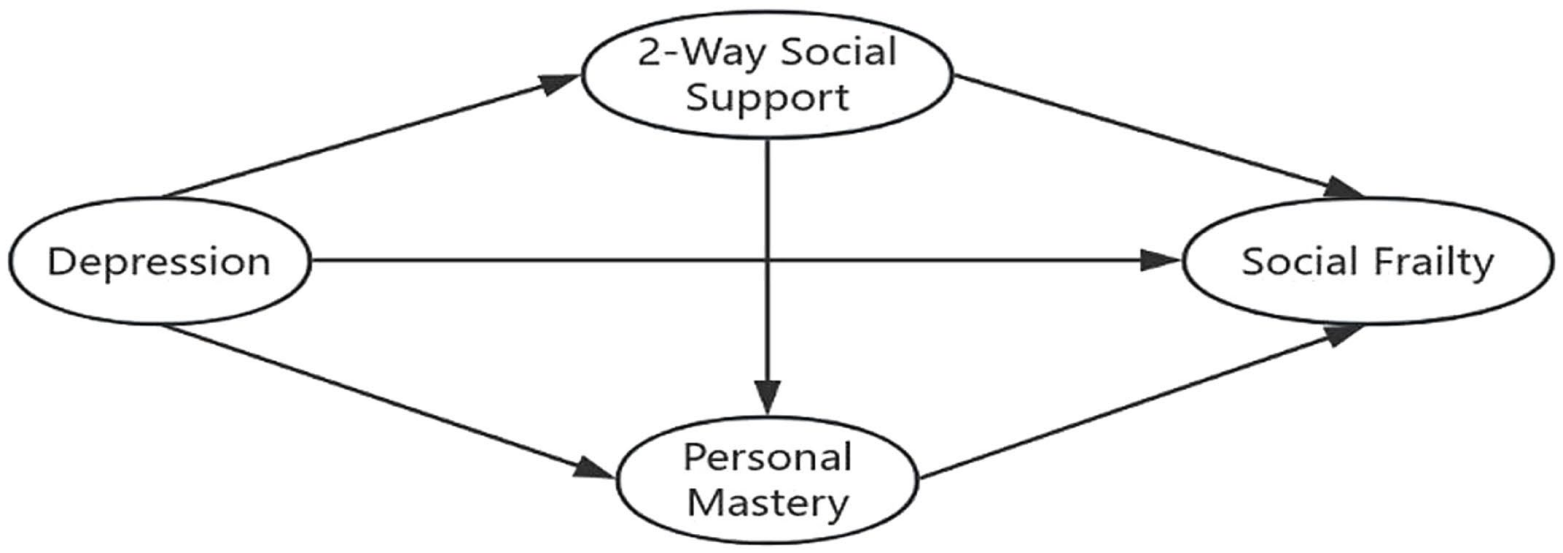
A theoretical model of the chain mediation effect.

This study is grounded in Stress Theory, a psychological framework that conceptualizes stress as an intermediary process linking stressors to stress outcomes. Within this model, cognitive appraisal and social support function as key mediating variables. In the context of this study, personal mastery represents an individual’s cognitive reappraisal following exposure to stressors. For elderly MHD patients, stressors may include factors such as advanced age, cultural adaptation, and marital status, which can contribute to the development of depressive symptoms. Drawing from both theoretical perspectives and prior literature, the study posits that personal mastery (cognitive appraisal) and bidirectional social support (social support) mediate the relationship between negative emotions (stressors) and social frailty (stress outcome). Furthermore, existing evidence suggests that bidirectional social support can influence personal mastery (24), indicating a potential interactive pathway between these mediators. Based on the literature and theoretical rationale, a chain mediation model was constructed, as illustrated in Figure 1. The following research hypotheses were proposed: (1) depression directly predicts social frailty in elderly MHD patients; (2) bidirectional social support partially mediates the relationship between depression and social frailty; (3) personal mastery partially mediates the relationship between depression and social frailty; and (4) bidirectional social support and personal mastery jointly serve as multiple mediators in the relationship between depression and social frailty.

## Methods

### Participants

A convenience sampling method was used to recruit 248 elderly patients undergoing maintenance hemodialysis (MHD) from blood purification centers in two tertiary hospitals in Wuhan, China, between August and December 2024. Inclusion criteria were as follows: (1) undergoing MHD for at least 3 months; (2) aged 60 years or older; (3) the ability to understand and independently respond to questionnaires; and (4) provision of informed consent and voluntary participation. Exclusion criteria included: (1) comorbid severe physical or psychiatric disorders; (2) presence of major systemic diseases or malignancies; (3) having received psychological counseling within the past 3 months; and (4) audiovisual impairments that hindered participation. All the scales in this study were measured simultaneously through a single questionnaire survey.

### Measures

#### General information about the patients

A self-designed questionnaire was used to collect demographic and clinical information, including gender, age, education level, marital status, personal monthly income, dialysis duration, and dialysis frequency.

#### Geriatric Depression Scale 5

The GDS-5 was developed and validated by Hoyl et al. (25), and consists of five items selected from the original 15-item Geriatric Depression Scale (GDS-15) based on their clinical relevance and diagnostic value. The scale is designed to assess depressive symptoms in older adults. Each item is answered with “yes” or “no,” and responses are scored as either 0 or 1, yielding a total score ranging from 0 to 5. A score of ≥2 indicates the presence of depressive symptoms, with higher scores reflecting greater severity. In the present study, the scale demonstrated acceptable internal consistency, with a Cronbach’s alpha of 0.74.

#### Social Vulnerability Index

The SVI was originally developed by Andrew et al. (26) and later translated into Chinese by Bai Hui-Qiong et al. (27). It is primarily used to assess social frailty among older adults. The instrument comprises 32 items across seven dimensions: access to information, living situation, sense of control over life, socioeconomic status, life satisfaction, activity level, and social support. Items are scored using dichotomous or trichotomous formats. The total score ranges from 0 to 32, and the index score is calculated by dividing the total score by the number of items, yielding a value between 0 and 1. A total score ≥0.5 indicates moderate to severe social frailty. Higher SVI scores reflect greater levels of social frailty. In the present study, the scale demonstrated acceptable internal consistency, with a Cronbach’s alpha coefficient of 0.76.

#### Personal Mastery Scale

The PMS was originally developed by Pearlin et al. (28), and subsequently revised and validated for the Chinese population by Yu Yibing et al. (29). It comprises seven items within a single dimension, measured using a 5-point Likert scale ranging from “not at all” (1) to “completely” (5). Total scores range from 7 to 35, with higher scores indicating a greater sense of personal mastery. In the present study, the scale demonstrated excellent internal consistency, with a Cronbach’s alpha coefficient of 0.926.

#### The Brief 2-Way Social Support Scale

The scale was originally developed by Obst et al. (30), and later revised and validated for use in Chinese populations by Cui Yu et al. in 2019 (31). It consists of 12 items across four dimensions: receiving emotional support, providing emotional support, receiving instrumental support, and providing instrumental support. Each item is rated on a 5-point Likert scale ranging from “not at all” (1) to “completely” (5), with total scores ranging from 12 to 60. Higher scores reflect greater levels of bidirectional social support. In this study, the scale demonstrated excellent internal consistency, with a Cronbach’s alpha coefficient of 0.94.

#### Data collection

The sample size for cross-sectional surveys depends on the relevant variables under investigation. Kendall suggests a sample size of 5 to 10 times the number of variables. This study includes 30 independent variables, necessitating a minimum sample size of *N* = 30 × (5~10) = 150 ~ 300 cases. Accounting for a 10% attrition rate, the sample size ranges from 165 to 330 cases. The final sample size for this study was 248 cases. Data were collected through face-to-face interviews conducted during maintenance hemodialysis sessions, specifically between 1 h after treatment initiation and 1 h before completion. Prior to participation, the purpose, significance, instructions, and precautions related to the study were explained to each patient. Researchers administered the questionnaires individually and assisted participants as needed. Each survey required approximately 15–20 min to complete. Upon completion, the responses were reviewed immediately to identify and correct any omissions or errors through direct communication with the participants. A total of 260 questionnaires were distributed, and 248 valid responses were collected, yielding a response rate of 95.4%.

#### Statistical analyses

All statistical analyses were conducted using SPSS version 22.0 and AMOS version 28.0. Categorical variables were presented as frequencies and percentages (*n*, %), while continuous variables with normal distributions were reported as means and standard deviations ( $\bar{x}\pm s$ ). Pearson correlation analysis was performed to examine the relationships among variables. Structural Equation Modeling (SEM) was conducted using AMOS 28.0 to evaluate the hypothesized pathways. Model fit was assessed using the following indices: relative chi-square (*x^2^/df*<5), Root Mean Square Error of Approximation (RMSEA < 0.08), Goodness-of-Fit Index (GFI > 0.90), Normed Fit Index (NFI > 0.90), Comparative Fit Index (CFI > 0.90), and Incremental Fit Index (IFI > 0.90). Using the bootstrapping method to repeatedly sample 2000 times to test the mediating effect of social frailty can effectively reduce sampling variation errors, with *p* < 0.05 indicating statistically significant differences.

Based on prior literature and clinical knowledge, we incorporated key covariates such as age, education, marital status, income, and dialysis duration into the mediational model to control for confounding bias in the mediational pathways. Furthermore, all core variables (including GDS-5, SVI, Brief 2-Way Social Support Scale, and PMS) were measured using established scales with validated reliability and validity to ensure data accuracy.

## Results

### Common method bias test

To evaluate the potential for common method bias resulting from the use of multiple self-report instruments, Harman’s single-factor test was conducted. The results revealed that 10 factors had eigenvalues greater than 1, and the variance explained by the first factor was 31.59%, which is below the critical threshold of 40%. These findings suggest that common method bias was not a significant concern in this study.

### Comparison of social frailty scores among elderly hemodialysis patients with varying characteristics

Among the 248 elderly maintenance hemodialysis (MHD) patients, the majority were female (51.2%), aged between 60 and 88 years (67.23 ± 6.92), had an education level of junior high school or below (58.1%), were married (75.8%), reported a monthly income below 5,000 yuan (77.4%), had undergone dialysis for more than 3 years (64.1%), and received dialysis thrice weekly (54.4%). Statistically significant differences in social frailty scores were observed among elderly MHD patients based on age, education level, marital status, personal monthly income, and dialysis duration (*p* < 0.05). In contrast, differences related to gender and dialysis frequency were not statistically significant (*p* > 0.05), as shown in Table 1.

**Table 1:** General demographics of elderly MHD patients (*N* = 248).

Variable	Categories	*n*, %	$\bar{x}\pm s$	*t/F*	*p-*value
Gender	Male	121 (48.8)	0.37 ± 0.19	2.078	0.039
	Female	127 (51.2)	0.42 ± 0.17		
Age	60 ~ 69 years	160 (64.5)	0.37 ± 0.18	5.259	<0.01
	70 ~ 79 years	74 (29.8)	0.45 ± 0.20		
	≥80 years	14 (5.7)	0.39 ± 0.13		
Education	Junior high school or below	144 (58.1)	0.47 ± 0.18	21.857	<0.01
	High school or technical secondary school	71 (28.6)	0.32 ± 0.14		
	College or above	33 (13.3)	0.26 ± 0.12		
Marital status	Married	188 (75.8)	0.35 ± 0.16	6.847	<0.01
	Divorced/Widowed	60 (24.2)	0.52 ± 0.21		
Personal monthly income	<696 USD	192 (77.4)	0.42 ± 0.19	4.70	<0.01
	≥ 696 USD	56 (22.6)	0.30 ± 0.14		
Dialysis duration	<1 year	30 (8.1)	0.37 ± 0.13	7.197	<0.01
	1 ~ 3 years	59 (23.8)	0.32 ± 0.16		
	>3 years	159 (64.1)	0.42 ± 0.20		
Dialysis frequency	Twice a week.	61 (24.6)	0.43 ± 0.19	2.479	0.086
	Three times a week.	135 (54.4)	0.37 ± 0.17		
	Others	52 (21.0)	0.42 ± 0.21		

### Depression, personal mastery, two-way social support, and social frailty scores

The mean depression score among elderly maintenance hemodialysis (MHD) patients was (1.91 ± 1.67), indicating a low level of depressive symptoms, with 53.6% of patients meeting criteria for depression. The two-way social support score averaged (38.49 ± 8.43), reflecting a moderate level. The scores for received and provided social support were (19.27 ± 4.27) and (19.23 ± 4.35), respectively. The personal mastery score was (21.51 ± 4.95), indicating a moderate level. The social frailty score was (0.49 ± 0.19), indicating moderate impairment, as detailed in Table 2.

**Table 2:** Depression, personal mastery, two-way social support, and social frailty scores (*N* = 248).

Variables	Range of score	Score ( $\bar{x}\pm s$ )	Scoring rate (%)
Depression	0 ~ 5	1.91 ± 1.67	23.80
Two-way social support	12 ~ 60	38.49 ± 8.43	64.15
Personal mastery	7 ~ 35	21.51 ± 4.95	61.46
SVI	0 ~ 1	0.49 ± 0.19	49.00

Scoring rate: Actual score/maximum theoretical score×100%.

### Correlation analysis of depression, personal mastery, two-way social support and social frailty in elderly hemodialysis patients

Pearson’s correlation analysis revealed a significant positive correlation between depression and social frailty (*r* = 0.716, *p* < 0.01), while personal mastery and two-way social support were significantly negatively correlated with social frailty (*r* = −0.721 and *r* = −0.760, respectively *p* < 0.01). Details are presented in Table 3. The permissible errors of the variables ranged from 0.376 to 0.456, and the variance inflation factors (VIF) ranged from 2.19 to 2.66, all below the threshold of 3, indicating the absence of multicollinearity among the variables.

**Table 3:** Correlations between observed variables (r).

Variable	SVI	Information Acquisition	Living status	Sense of control	Socio-economic status	Life satisfaction	Activity status	Social support
Depression	0.716^**^	0.143^*^	0.246^**^	0.549^**^	0.481^**^	0.498^**^	0.709^**^	0.517^**^
Personal mastery	−0.721^**^	−0.265^**^	−0.181^**^	−0.462^**^	−0.628^**^	−0.633^**^	−0.695^**^	−0.487^**^
Two-way social support	−0.760^**^	−0.194^**^	−0.322^**^	−0.612^**^	−0.537^**^	−0.525^**^	−0.688^**^	−0.539^**^
Receive emotional support	−0.703^**^	−0.150^*^	−0.378^**^	−0.600^**^	−0.452^**^	−0.396^**^	−0.650^**^	−0.472^**^
Giving emotional support	−0.733^**^	−0.210^**^	−0.399^**^	−0.630^**^	−0.469^**^	−0.403^**^	−0.664^**^	−0.493^**^
Accept tool support	−0.588^**^	−0.147^*^	−0.181^**^	−0.451^**^	−0.457^**^	−0.581^**^	−0.518^**^	−0.451^**^
Giving tool support	−0.549^**^	−0.150^*^	−0.141^*^	−0.395^**^	−0.439^**^	−0.489^**^	−0.499^**^	−0.408^**^

*p < 0.05; **p < 0.01.

### A model test of the relationship between depression, personal mastery, two-way social support and social support in elderly hemodialysis patients

**Figure 2:**
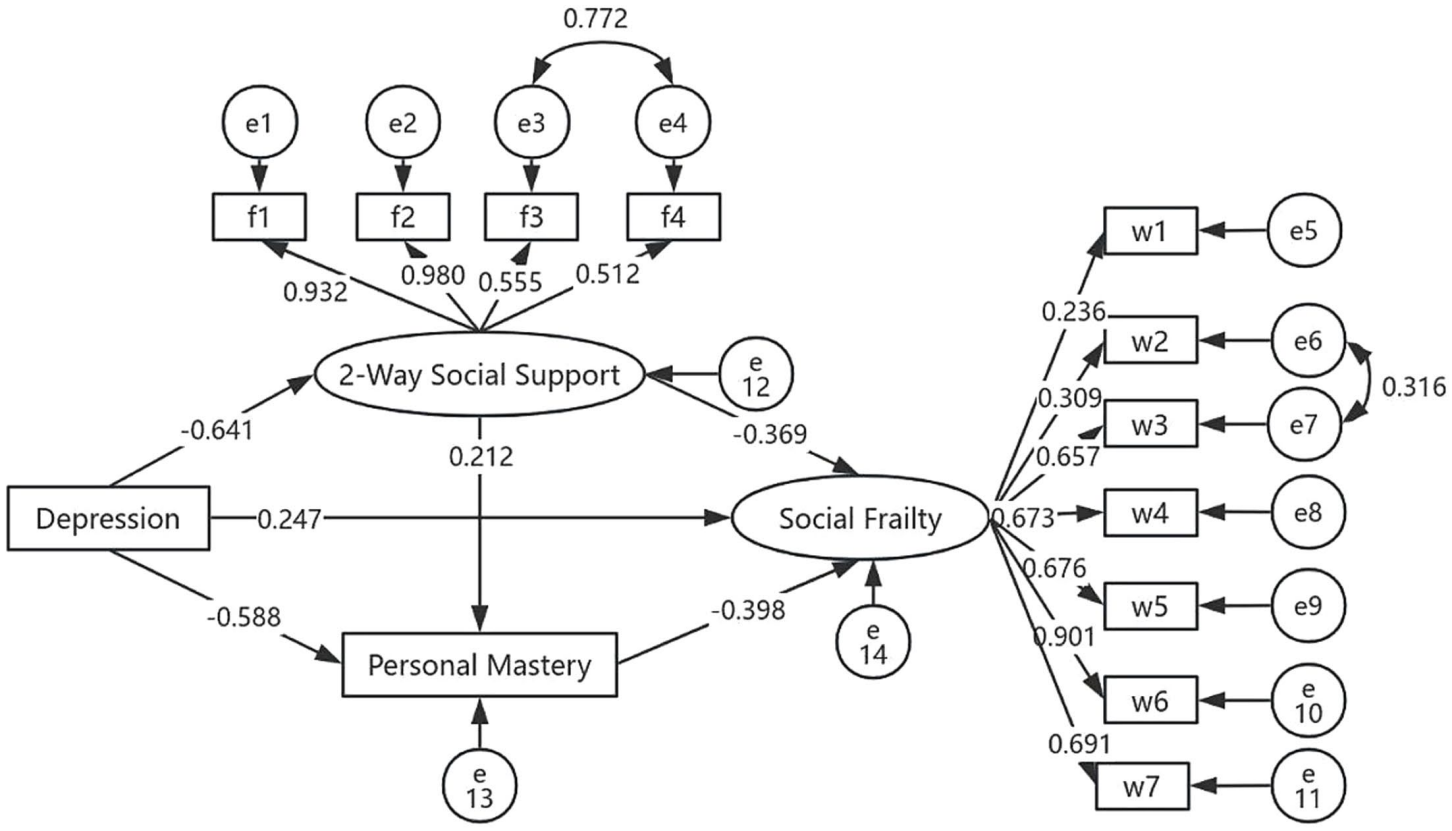
Finalized structural model (*N* = 248). All factor loadings are standardized.

The correlation analysis revealed significant associations among depression, bidirectional social support, personal mastery, and social frailty, which fulfilled the prerequisite for testing mediation effects. In this study, an initial structural equation model (SEM) was constructed, with depression as the independent (observed) variable, bidirectional social support and personal mastery as mediators, and social frailty as the dependent variable. The initial model exhibited poor fit and was subsequently revised. The revised model demonstrated acceptable fit indices: *x^2^/df* = 4.003, RMSEA = 0.08, GFI = 0.908, NFI = 0.918, CFI = 0.917, and IFI = 0.918, indicating a good model fit. The finalized model is presented in Figure 2.

The bootstrap method with 2000 resamples was employed to assess the mediating effects on social frailty. The analysis revealed that the multiple mediating effects of bidirectional social support and personal mastery were statistically significant. Both variables served as partial mediators between depression and social frailty among elderly MHD patients. Specifically: (1) the total indirect effect accounted for 67.96% of the total effect; (2) the direct effect accounted for 32.04%; (3) the indirect effect via “depression → two-way social support → social frailty” accounted for 30.61%; (4) the indirect effect via “depression → personal mastery → social frailty” accounted for 30.35%; (5) the chain mediation effect via “depression → two-way social support → personal mastery → social frailty” accounted for 7.00%; and (6) the total effect of depression on social frailty was 0.771, while the inclusion of bidirectional social support and personal mastery in the model yielded a standardized direct effect of 0.247, as detailed in Table 4.

**Table 4:** Effect analysis of factors influencing social frailty in elderly hemodialysis patients.

Structural path	Standard coefficients	SE	95% CI	Effect size
Total effect	0.772	0.016	0.719 ~ 0.821	100%
Direct effect	0.247^**^	0.007	0.141 ~ 0.348	32.04%
Total indirect effect	0.525^**^	0.012	0.439 ~ 0.624	67.96%
Depression→two-way social support→social frailty	0.237^**^	0.006	0.155 ~ 0.329	30.61%
Depression→personal mastery→social frailty	0.234^**^	0.006	0.149 ~ 0.332	30.35%
Depression→two-way social support→personal mastery→social frailty	0.054^**^	0.002	0.025 ~ 0.094	7.00%

**p < 0.01.

## Discussion

The aim of this study was to explore the relationship between depression and social frailty in the elderly maintenance hemodialysis (MHD) population, with a particular focus on the mediating effects of bidirectional social support and personal mastery. Our analysis revealed three principal findings: First, social frailty was found to be more severe among older MHD patients. Second, depression was significantly positively correlated with social frailty. Third, bidirectional social support and personal mastery were identified as sequential mediators in the relationship between depression and social frailty. The present study revealed a moderate level of social frailty, higher than that reported in a French community-dwelling elderly population (0.38 ± 0.18) (32). This disparity may be attributed to age-related declines in physical function (33) and changes in family structure (34), both of which reduce opportunities for social engagement. The requirement for two to three” dialysis sessions per week significantly limits time available for social activities, making it difficult for patients to maintain employment or engage in regular social interactions, thereby intensifying unmet social needs and aggravating social frailty. Therefore, treatment goals should extend beyond alleviating clinical symptoms and prolonging survival to include enhancing patient’ social functioning, addressing unmet social needs, and preventing the progression of social frailty.

The results demonstrated that depression exerted a direct positive effect on social frailty among elderly MHD patients. Specifically, greater severity of depressive symptoms was associated with an increased risk of social frailty, consistent with the findings of Chen et al. (35). Depressive states are associated with elevated levels of chronic inflammatory markers, which can directly accelerate muscle catabolism and physical functional decline (36). Additionally, depression directly or indirectly reduces patients’ motivation for physical and social engagement (37), exacerbates the loss of social roles, and hinders the re-establishment of social networks, thereby increasing the risk of social frailty. Therefore, early identification of depressive symptoms, regular assessment of their causes and onset, and timely clinical intervention are essential. Patients should be encouraged to express their emotions and participate in peer-based activities aimed at fostering emotional and material support, thereby improving psychological well-being and promoting social integration.

Bidirectional social support was found to partially mediate the relationship between depression and social frailty. This finding suggests that depressive symptoms may influence social frailty indirectly through the mechanism of bidirectional social support. Patients with depression often exhibit reduced willingness to interact with family and friends, resulting in lower levels of social support. According to the stress-buffering model, social support functions as a protective factor that can mitigate the impact of stressors on an individual’s physical and mental health (38). Moreover, bidirectional social support is negatively associated with social frailty (39), suggesting that individuals with higher levels of support are more capable of accessing and utilizing available resources, fostering positive social interactions, and are less susceptible to social frailty. When facing stressful events, patients may reduce social withdrawal caused by dialysis-related stress through receiving emotional and instrumental support, thereby enhancing social participation. Patients who actively provide social support can enhance their sense of self-worth and foster meaningful social contributions, thus promoting a virtuous cycle of interaction and reciprocity within their social networks.

Personal mastery was found to partially mediate the relationship between depression and social frailty. This finding indicates that individuals with depressive symptoms may influence social frailty through their level of personal mastery. According to the limited resource model of self-control (40), individuals possess finite cognitive and emotional resources, which can be temporarily mobilized but are gradually depleted under sustained external stressors, leading to psychological resource scarcity. A personal sense of control serves as an essential internal coping resource. Patients with a high level of personal mastery exhibit stronger coping mechanisms when confronted with dialysis-related stress. They are more adept at reframing problems into actionable coping strategies, actively participating in treatment, and engaging in social activities. These behaviors enhance illness management and emotional regulation, thereby reducing depressive symptoms, minimizing social withdrawal, and ultimately lowering the risk of social frailty.

Bidirectional social support and personal mastery demonstrated a chain-mediating effect between depression and social frailty. These findings suggest that depression may indirectly reduce social frailty by enhancing personal mastery through bidirectional social support. Higher levels of bidirectional social support were associated with greater emotional and instrumental support from family and friends, which enhanced patients’ confidence in managing their health and subsequently increased their sense of personal mastery. Providing support to others further reinforces patients’ self-worth, strengthens their intrinsic motivation, and enhances their sense of control, thereby reducing the risk of social frailty. Prolonged dialysis duration and the accumulation of uremic toxins may exacerbate depressive symptoms, adversely affecting both quality of life and the likelihood of social frailty. Therefore, the findings of this mediation model highlight the urgent need for mental health interventions that not only target the direct outcomes of social frailty, but also address the underlying mediating mechanisms.

In summary, healthcare professionals should not only address patients’ depression but also emphasize the enhancement of patients’ bidirectional social support and sense of personal mastery. Enhancing these factors can help maintain psychological well-being and mitigate the development of social frailty. Bidirectional social support and personal mastery are critical factors in alleviating depression. If depression is not treated promptly, it may affect the patient’s treatment compliance, dialysis adherence, and quality of life management (14). In recent years, associations have been identified between mental health and biochemical indicators such as serum phosphorus levels. This has opened up new avenues for improving patients’ quality of life through psychological and behavioral interventions, and underscores the importance of interdisciplinary collaboration between nephrology and psychology (41). Based on research findings, we have established a clinical practice framework encompassing screening, referral, intervention, and follow-up. First, routine psychosocial assessments are implemented in dialysis centers using standardized scales (e.g., GDS-5, SVI) for preliminary symptom identification. Second, multidisciplinary tiered referrals are conducted based on screening results. Patients with moderate to severe depression are referred to psychiatric services. Individuals with insufficient social support are referred to medical social workers to initiate a “family-peer” support program. Individuals with low perceived self-efficacy should be enrolled in healthcare-led self-management education programs. During the intervention phase, cognitive behavioral therapy (CBT) and pharmacotherapy should be employed to address depressive symptoms and enhance emotional regulation abilities (42). Concurrently, active encouragement to participate in social activities should be provided, fostering psychological fulfillment and well-being through supporting others to strengthen bidirectional social support. Guide older adults to cultivate positive emotions, embrace self-regulation, and strengthen confidence in recovery. Leverage family and peer resources to conduct supportive activities, enhancing their sense of control over disease management and environmental coping. Finally, conduct regular follow-ups to dynamically assess intervention effectiveness. Reassess depression and social frailty every three to 6 months, dynamically adjust interventions based on follow-up results, and integrate this model into patients’ long-term chronic disease management systems to ensure sustained program efficacy.

## Conclusion

In summary, the mechanism by which depression, personal mastery, and bidirectional social support influence social frailty can be understood as a dynamic interplay between internal psychological resources and external environmental resources. Depressed patients often experience a depletion of resources manifested as low mood, diminished motivation, and reduced energy, which directly exacerbates social frailty. Conversely, personal mastery regulates internal psychological resources, while bidirectional social support indirectly mitigates social frailty by providing both material and emotional resources. This study found that bidirectional social support is significantly associated with social frailty. Moreover, both bidirectional social support and sense of personal mastery partially mediated the relationship between depression and social frailty in elderly maintenance hemodialysis (MHD) patients, with the mediating effect of bidirectional social support being significantly stronger than that of other mediators. The positive association between depression and social frailty can be attenuated by enhancing levels of bidirectional social support and personal mastery. This study validated the underlying mechanism linking depression and social frailty and extended previous research findings. Furthermore, it provides a theoretical foundation for developing intervention programs that prioritize personal mastery as the core component, bidirectional social support as the framework, and depression management as the fundamental basis. Nevertheless, this study has several limitations: (a) its cross-sectional design precludes determination of causality, warranting longitudinal studies for further exploration; (b) the relatively small sample size limits generalizability, thus future research should adopt a large-sample, multicenter approach; (c) the exclusive use of quantitative methods limits in-depth understanding of variable relationships, suggesting that mixed-methods studies could be beneficial to comprehensively explore the social phenomena involved.

## Data Availability

The datasets presented in this study can be found in online repositories. The names of the repository/repositories and accession number(s) can be found in the article/Supplementary material.
